# Parliament and the Profession

**Published:** 1918-08-10

**Authors:** 


### Parliament and the Profession.

### Bombing of Etaples Hospital.

COLONEL WEDGWOOD asked the Under-Secretary of State for War if he is aware that the German Government are now justifying the bombing of the Etaples Hospital on the night of May 19-20 by the allegation that the Red Cross was not shown; and whether he would inquire into and expose this statement. Mr. Macpherson said he was aware of the allegation, and he had seen in a German newspaper photographs published with the intention of proving that the Red Cross sign did not exist on May 21, but did exist in large numbers on May 27. German photographs of this nature, as there is reason to know, are never conclusive. In any case the hospitals were bombed on the night of May 31-June 1 in spite of the Red Cross signs, which German evidence shows to have been in existence on May 27. Mr. Macpherson said that he was personally satisfied that the Red Cross sign was shown on May 19.

### Small-pox during 1917.

MR. HAYES FISHER informed Mr. Chancellor that there were seven cases of small-pox notified in England and Wales in the year 1917. In the latest year for which complete figures are available, 50.1 per cent. of the infants born were successfully vaccinated.

### Gas-Mask Illness.

THE Under-Secretary of State for War was asked if he will furnish a return showing the number of men suffering from trench gums, a complaint alleged to be due to the combination of a nose-clip and mouth-piece, especially the latter, of the small box respirator attached to the gas-mask at present in use in the field. Mr. Macpherson said that a special investigation, of which this subject will form a part, has recently been commenced at the Royal Army Medical College, and it is hoped that this will result in valuable information being obtained upon the prevalence and prevention of this condition.

